# Effect of a Multicomponent Program on the Prevention of Cognitive Decline and Dementia

**DOI:** 10.1192/j.eurpsy.2026.11579

**Published:** 2026-07-31

**Authors:** C. Gameiro, P. Varandas, B. Lopes, B. Freitas, C. Pombo

**Affiliations:** 1 Aging and Dementia; 2 Clinical Management, Sisters Hospitallers, Lisboa, Portugal

## Abstract

**Introduction:**

Multidomain programs have demonstrated efficacy in preventing cognitive decline and dementia. Additionally, technological solutions show potential for cognitive interventions and the promotion of healthy aging. This study investigated the implementation of an innovative multidomain program in a Portuguese community setting, inspired by international multidomain models and supported by technological interventions.

**Objectives:**

To evaluate the effect of a multidomain program on adherence, satisfaction, and outcomes in cognitive, affective, social, and quality of life domains.

**Methods:**

The program consisted of 26 sessions, held twice per week, combining robot-assisted therapy, augmented reality, computerized cognitive training, and psychoeducational sessions. Sociodemographic data and adherence records were collected, and a multidimensional assessment battery was administered before and after the intervention, along with a satisfaction questionnaire.

**Results:**

The study included 504 older adults (mean age = 76 ± 9.47 years), mostly women (80.36%). Significant improvements were observed in all assessed domains (p < 0.001): cognitive (+2.6 points ACE-R), affective (–1.1 points GDS-15), social (+3.9 points ESSS), and quality of life (+1.2 points WHOQOL-BREF). Adherence was 89%, and satisfaction was 98%.

**Conclusions:**

The program was effective, as evidenced by the multidimensional health gains achieved. The integration of innovative technological interventions, combined with a multidomain approach, appears promising for the prevention of cognitive decline. Future studies with control groups could strengthen the robustness and sustainability of these results.

**Disclosure of Interest:**

None Declared

